# Investigation of the value of hematological biomarkers in the clinical differential diagnosis of IgG4-RD

**DOI:** 10.55730/1300-0144.5629

**Published:** 2023-03-27

**Authors:** Hazan KARADENİZ, Aslıhan AVANOĞLU GÜLER, Rıza Can KARDAŞ, Muzaffer KARADENİZ, Hatice PAŞAOĞLU, Hamit KÜÇÜK, Berna GÖKER, Abdurrahman TUFAN, Mehmet Akif ÖZTÜRK

**Affiliations:** 1 Division of Rheumatology, Department of Internal Medicine, Faculty of Medicine, Gazi University, Ankara, Turkey; 2 Department of Cardiology, Faculty of Medicine, Kızılcahamam State Hospital, Ankara, Turkey; 3 Department of Biochemistry, Faculty of Medicine, Gazi University, Ankara, Turkey

**Keywords:** Systemic immune-inflammation index, systemic inflammation response index, neutrophil-lymphocyte ratio, platelet lymphocyte ratio, IgG4-related disease

## Abstract

**Background/aim:**

IgG4-related disease (IgG4-RD) is a systemic fibroinflammatory disease whose pathogenesis has not been completely elucidated. Due to the novelty and complexity of the diagnostic criteria, it is difficult to distinguish from the diseases included in the differential diagnosis without tissue biopsy. This study aimed to discover new biomarkers that can help for disease diagnosis and its differential diagnosis by reviewing the relationships between neutrophil-lymphocyte ratio (NLR), platelet lymphocyte ratio (PLR), systemic immune-inflammation index (SII), and systemic inflammation response index (SIRI).

**Materials and methods:**

Thirty IgG4-RD, 38 granulomatous polyangiitis (GPA), and 46 sarcoidosis patients presenting to the Rheumatology Clinic meeting the criteria of 2019 American College of Rheumatology, 2012 International Chapel Hill and 1999 American Thoracic Society meeting, respectively, and 27 healthy control subjects were included. We collected data on complete blood count with automated differential values including NLR, PLR, SII, and SIRI.

**Results:**

The SII and PLR values were significantly higher in patients with IgG4-RD compared to healthy controls, (SII median (min-max) 572 (102–5583) vs. 434 (172–897), PLR median (min-max) 130 (56.8–546) vs. 104 (57.5–253) p < 0.001). SII value was found to have a significant positive correlation with CRP in IgG4-RD disease (r = 0.371; p = 0.043). While SII, SIRI, NLR, PLR parameters were not significant between the IgG4-RD and sarcoidosis groups, SII, SIRI, NLR, PLR were significantly higher in patients with GPA than in IgG4-RD patients (p < 0.001).

**Conclusion:**

This is the first study to review the SII, SIRI, NLR, and PLR in IgG4-RD. The obtained results suggest that the SII could be used as a new tool, for differential diagnosis and activity of the IgG4-RD.

## 1. Introduction

IgG4-related disease (IgG4-RD) is a systemic fibroinflammatory disease, in which T cytotoxic cells and plasmablasts play a prominent role **[1]**. IgG4-RD is frequently detected incidentally during the histopathological or radiological examination of a tissue **[2,3]**. Diagnosis is established via combination of clinical, radiological, laboratory and pathologic characteristics of disease. Prior to the 2019 American College of Rheumatology (ACR)/European League Against Rheumatism (EULAR) diagnostic criteria, there was an identification criterion based on serum IgG4 levels and histopathological IgG4/IgG+ positivity. However, it was realized that serum IgG4 levels can be found to be normal in most IgG4-RD patient subtypes such as retroperitoneal fibrosis. For this reason, the former classification criteria are no longer used **[4]**. According to the newly defined criteria, even if the value of IgG4 in the patient’s serum and histopathology is negative, a total score of ≥20 after meeting the entry and exclusion criteria is enough to make the diagnosis of IgG4-RD **[5]**. IgG4-RD is a disease with chronic relapses, there is currently no objective marker to predict relapse as well as diagnosis. In some studies, serum IgG4 level was examined in terms of predicting relapse, but its levels were found to be normal during relapses **[6]**. In IgG4-RD, apart from the presence of serum IgG4, hypergammaglobulinemia, hyper-IgE, low titer positive antinuclear antibodies, eosinophilia, high CRP, rheumatoid factor, and hypocomplementemia can be seen in blood **[7]**. However, these parameters are not specific and may increase in many other diseases as well as IgG4-RD. Compared to the serum IgG4 level, level of serum plasmablast is a more valuable biomarker in the IgG4-RD but difficulty and complexity of its measurement limits its clinical use.

The differential diagnosis of IgG4-RD includes many diseases such as sarcoidosis and granulomatosis with polyangiitis (GPA) **[8–10]**. GPA and IgG4-RD have been reported to overlap, sometimes presenting as GPA with atypical features, such as pachymeningitis, orbital mass, or chronic periaortitis. In patients with bilateral hilar adenopathy, the clinical picture may imitate sarcoidosis. Thus, it is important to differentiate IgG4-RD from sarcoidosis and GPA, which are frequently encountered in clinical practice. In GPA and sarcoidosis, ANCA-IFA and angiotensin converting enzyme (ACE) might help in making diagnosis **[11,12]**. Yet, these markers can also be positive in IgG4-RD. Therefore, there is still no objective marker to assess the diagnosis, differential diagnosis, and prognosis of IgG4-RD. Recently, complete blood count parameters suggested to have diagnostic and prognostic value in many inflammatory diseases **[13]**. These inflammatory indices rapidly yield results since they are evaluated during routine blood count. We wanted to study simple tests, which may help in the determination of diagnosis and estimation of prognosis, such as systemic inflammatory index (SII), systemic inflammatory response index (SIRI), platelet/lymphocyte ratio (PLR), neutrophil/lymphocyte ratio (NLR) in IgG4-RD patients and, if possible, to find a cutoff value for disease.

## 2. Materials and methods

Thirty IgG4-RD (13 female, 17 male; mean age 55.4 ± 14), 38 GPA (22 female, 16 male; mean age 48.2 ± 15.2), and 46 sarcoidosis (32 female,14 male 53.5 ± 12.4) patients presenting to the rheumatology clinic, and 27 healthy controls (15 female, 12 male; mean age 42.6 ± 14.1) were included. Of the patients with IgG4-RD, GPA and sarcoidosis, 16.6% (n = 5), 21.7% (n = 10), 21% (n = 8) were newly diagnosed. Patients with cases with isolated upper respiratory tract disease, orbital involvement, salivary gland involvement, lung and lymph node involvement were included. We excluded patients <18 years of age and those with another disease that could reason elevate SII, SIRI, NLR, and PLR values: i.e. patients with any type of malignancy, haematological diseases, serious cardiac disorder (advanced cardiac failure, cardiac diseases causing severe clinical symptoms, unstable arrhythmia, myocardial infarction in past ninety days), hepatic enzyme elevations due to any cause, different chronic respiratory diseases (bronchiectasis, interstitial lung disease, overlap syndrome) systemic diseases and COVID-19. Patients fulfilled the 2019 ACR for IgG4-RD, the 2012 revised International Chapel Hill Consensus Conference classification criteria for GPA, and the 1999 American Thoracic Society classification criteria for sarcoidosis **[5,14,15]**. Subgroups of GPA and sarcoidosis with findings of especially pulmonary failure/haemorrhage, renal failure, and neurological involvement were excepted from the study.

Individuals were examined and evaluated by an experienced rheumatologist. Laboratory, treatment, clinical, and demographic data were collected from patients upon admission. Demographic data included age, gender, and smoking habits (current/former/ever). The smoking index was calculated with years of tobacco use × number of cigarettes smoked per day. Laboratory tests were collected from all patients upon hospitalization before the start of medications. They included; complete blood count (CBC), erythrocyte sedimentation rate (ESR), and C-reactive protein (CRP). CBC with automated differential counts included neutrophils, lymphocytes, platelets, and differential values. In the control group, CBC and differential values, serum biochemical tests, ESR, and CRP were measured. Neutrophil lymphocyte ratio (NLR) was calculated as the ratio of neutrophils to lymphocytes. Platelet lymphocyte ratio (PLR) was acquired by dividing the absolute number of platelets to lymphocytes **[16]**. The SII is provided by calculating (thrombocyte×neutrophil)/lymphocyte; the SIRI: is provided by calculating (monocyte×neutrophil)/lymphocyte **[17]**. The laboratory reference values of white blood cells, neutrophils, lymphocytes, and eosinophils were 4.2–10, 1.8–7.3, 1.5–4, and 0.05–0.35 (×) 103/uL, respectively. This study was approved by the ethics committee (approval no. 2020-549), conducted in accordance with the 1975 Helsinki Declaration. Written informed consents were obtained from all participants prior to enrolment into the study.

## 3. Statistical analysis

For statistical analyses, NCSS (Number Cruncher Statistical System) 2007 (Kaysville, Utah, USA) program was used. In the evaluation of data, descriptive statistical methods (mean, standard deviation, median, frequency, percentage, minimum, maximum) were utilized. Whether quantitative data were distributed normally was evaluated with the Shapiro-Wilk test and graphics. In the comparison of more than two groups between normally distributed quantitative parameters, one-way ANOVA analysis, and Bonferroni test evaluations were made. When quantitative parameters were not normally distributed, in the comparisons between more than two groups, the Kruskal-Wallis test and Dunn’s test were used. Chi-square or Fisher’s exact tests were used to compare categorical variables. In the evaluation of the relationship between quantitative variables, Pearson’s and Spearman’s correlation tests were utilized for correlation analyses. In order to determine predictive values for parameters, diagnostic screening tests (sensitivity, specificity, PPV, NPV) and ROC analyses were used distinguishing IgG4 patients from healthy subjects. The optimal cutoff values for the sensitivity and specificity of SIRI, SII, NLR, and PLR in predicting IgG4-RD were calculated by using Youden’s index. The p-value of <0.05 was considered statistically significant.

## 4. Results

**Figure 1:**
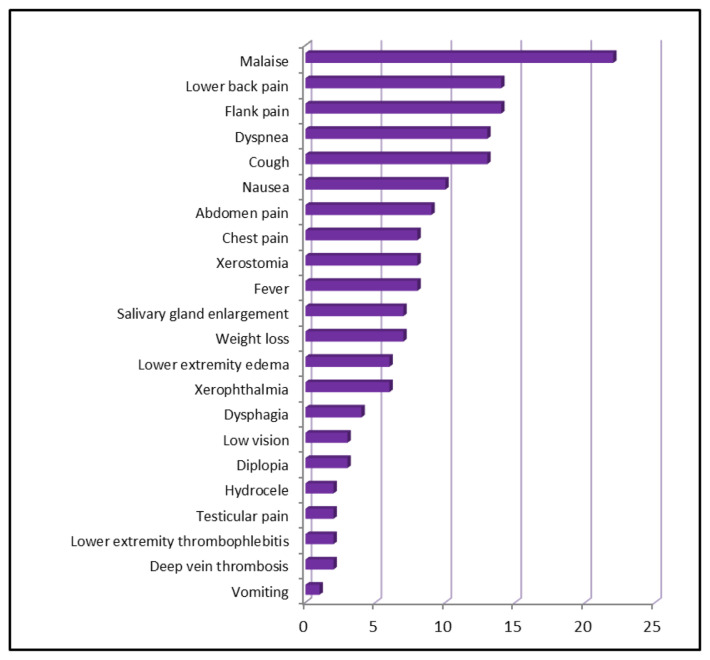
Distribution of symptoms in IgG4-RD.

Thirty IgG4-RD, 38 GPA, and 46 sarcoidosis patients were enrolled. Characteristics of the study patients and 27 healthy controls are depicted in Table 1. There were no significant differences between groups in terms of age and sex (p > 0.05). The mean age of the IgG4-RD group was 55.4 years, and most were male (56.7%). The smoking habit index was found to be significantly higher in the IgG4-RD group compared to other groups (p < 0.001). In the evaluation of symptoms of IgG4-RD cases, 73.3% (n = 22) was found to have malaise, 46.7% (n = 14) flank pain and, 46.7% (n = 14) lower back pain **(**Figure 1). Majority of patients (63.3%) had comorbid diseases. The most observed comorbidities were hypertension 33.3% (n = 10) and diabetes mellitus 6.7%, (n = 2), respectively. Radiologically, a space-occupying mass was detected in 69% (n = 20) of IgG4-RD patients, the most common type of involvement was being RPF (n = 11) at percentage of 37.9, followed by lung involvement 24.1% (n = 7). Lymph node involvement was evident in 37.9% (n = 11) of IgG4-RD cases. In the histopathological examinations, 70% of participants was found to have (n = 21) dense lymphoplasmacytic and 28.6% (n = 6) storiform fibrosis, and %4.8 (n = 1) obliterative phlebitis. In 20% of patients, (n = 6) dense lymphocytic infiltration and storiform fibrosis were coexisted **(**Table 2). ANA, ANCA and RF were positive in 13%, 20%, and 11.8% of patients, respectively. dsDNA, Sm, SSA and SSB antibodies were negative in all of the cases. In %60 of patients, serum IgG4 level was within normal limits, > 2–5-fold in 10% (n = 3), and > 5-fold in 10% (n = 3) of patients. In 30% (n = 9) of patients, histopathological evaluation of tissue specimen was inconclusive.

**Figure 2:**
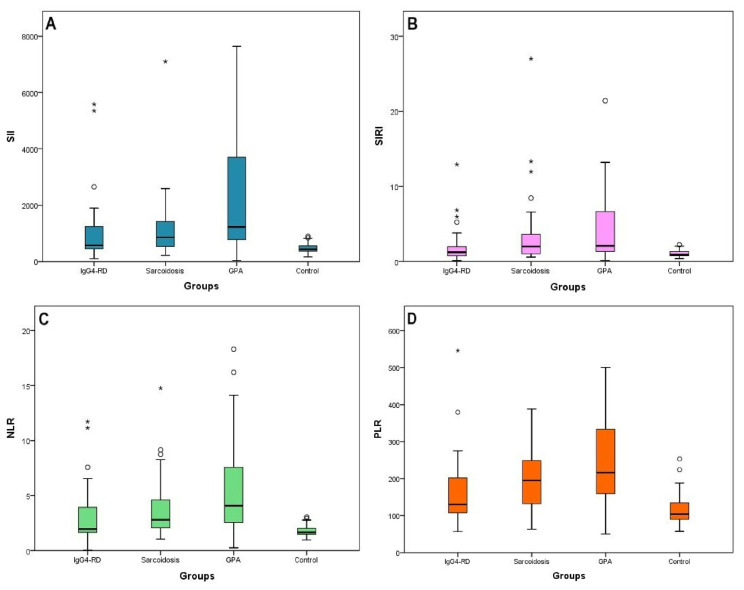
Distribution of SII (A), SIRI (B), NLR (C), PLR (D) values by groups.

**Figure 3:**
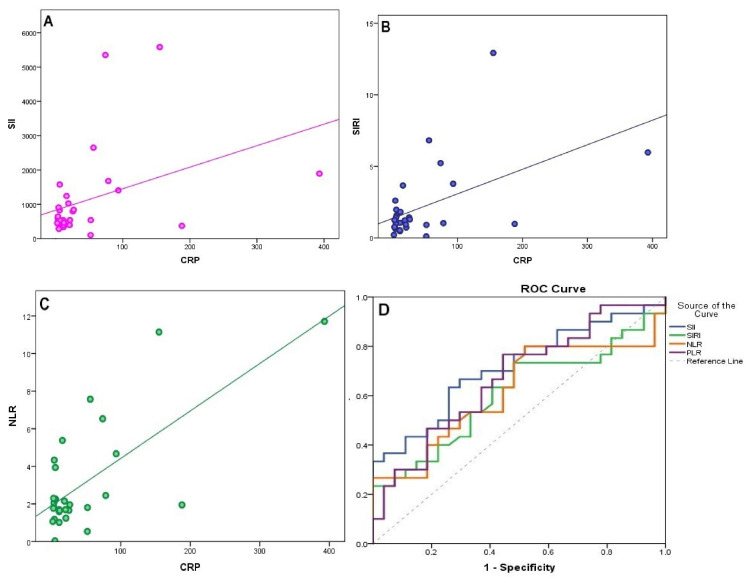
Correlation of CRP with SII (A), SIRI (B), NLR (C), and ROC curve analyses (D) in IgG4-RD. SII (AUC: 0.714, p: 0.006), SIRI (AUC: 0.606, p: 0.169), NLR (AUC: 0.617, p: 0.129), PLR (AUC: 0.670, p: 0.027).

In the comparison between groups, SII, SIRI, PLR and NLR levels were found to be significantly higher in GPA and sarcoidosis groups than in the control group, while SII and PLR values were found to be significantly higher in the IgG4-RD group **(**Table 3). SIRI and NLR values were higher in the IgG4-RD group than the control group, but did not reach statistical significance. SII, SIRI, NLR, and PLR values were found to be significantly higher in GPA group than IgG4-RD group **(**Figure 2A). SII, SIRI, NLR, and PLR values were found to be higher in sarcoidosis group than IgG4-RD group, but the difference was not statistically significant **(**Figure 2B). No difference was found between GPA and sarcoidosis groups with regard to inflammatory indices **(**Figure 2C). Lymphocyte values were found to be higher in IgG4-RD group than GPA, sarcoidosis, and control groups, whilst higher neutrophil values were found in the GPA group **(**Figure 2D). RDW value was found to be lowest in the IgG4-RD group, while there was no significant difference between groups with respect to MPV values. In correlation analysis, SII (r = 0.37; p = 0.04) **(**Figure 3A), SIRI r = 0.37; p = 0.04) **(**Figure 3B), and NLR (r = 0.37; p = 0.04) **(**Figure 3C) values were found to have positive correlation with CRP, which is the traditional disease activity marker of IgG4-RD disease. In IgG4-RD patients, cutoff value for SII was determined as ≥ 535 (×) 109/L (sensitivity 63.3%, specificity 74.1%, positive predictive value 73.1% and negative predictive value 64.5%). Similarly, cutoff value for PLR was established to be ≥108 (sensitivity 76.7%, specificity 55.6%, positive predict value 65.7% and negative prediction value 68.2%). For GPA and sarcoidosis patients, SII cut-off values were found as ≥718 (×) 109/L (sensitivity 86.4%, specificity 81.5%) and ≥ 537(×)109/L (sensitivity 76.1%, specificity 74.1%), respectively. In the SII ROC curve, area under the curve was found to be 71.4% with 6.8% standard error. The corresponding values were 60.6% and 7. 6%, respectively, for SIRI; 61.7% and 7.6%, for NRL, and 67%, and 7.2% for PRL. When indices were compared two by two, it was established that SII had a higher level of prediction of IgG4 than NRL: (p = 0.001). No significant difference was found between IgG4 predictive values of SII, SIRI, and PLR (p = 0. 085; p = 0.4; p > 0.05) **(**Figure 3D).

## 5. Discussion

IgG4-RD is a relatively new disease with limited diagnostic and prognostic biomarkers. Hematological indices such as PLR and NLR have been investigated and found to be beneficial in the diagnosis and follow-up of many rheumatologic diseases such as SLE, RA, and AS **[13,18]**. Recently, new indices such as SII and SIRI have been described in addition to NLR and PLR **[19]**. However, none of these markers have been investigated so far in IgG4-RD disease and their clinical utility. We found that SII and PLR values could distinguish IgG4-RD from healthy controls and additionally SII, SIRI, and NLR were correlated with the traditional activity marker CRP. We also found that SII was more sensitive than other indices for predicting the diagnosis of IgG4-RD. In addition, we also determined that inflammation was more severe in the GPA group than other groups, even in the absence of systemic involvement.

Both innate and adaptive immune systems play part in the pathogenesis of IgG4-RD. T lymphocytes constitute a significant percentage of lymphocytes found in the tissues of patients with IgG4-RD. Apart from T-cells, it has been identified in hypermutated plasmablasts in the tissues and blood of active IgG4-RD patients. General histopathology findings consist of small lymphocytes, mild to moderate eosinophilia, mature, immature plasma cells and dense lymphoplasmacytic infiltrates consisting of IgG4+ plasma cells. Lymphocytes, neutrophils, and monocytes act important role in general inflammation. During inflammation, a decrease in lymphocyte count is accompanied by an increase in neutrophil and platelet counts. Therefore, the proportions of these cells become an important diagnostic tool both in demonstrating the inflammatory state and in indirectly assessing the cell-mediated immune status. While we know the vital roles of neutrophils and lymphocytes in inflammation, we know less about the role of platelets in it **[20]**. Contrary to what is known, platelets have a very important role in the immune system due to their surface receptors that not only take part in coagulation and fibrinolysis but also recognize pathogens and immune complexes.

As mentioned above, inflammatory cells have a varying degree of role in the pathogenesis of IgG4-RD. However, evaluating them on their yields less information than the ratio between them. Therefore, inflammation has to be considered as a whole e.g., while no difference was found between the four groups in terms of platelet counts significant differences were found between groups for PLR and SII indices. All such indices, especially NLR and PLR are practical and cost-effective prognostic markers, which have long been used in various diseases. In malignancies, they were found to be high during disease activity and, decrease when the disease went into remission. In a study carried out on COVID-19, it was established that adverse prognostic factors such as ferritin, d-dimer and eosinophilia correlated positively with NLR and PLR markers. It was seen that these indices correlated not only with laboratory markers but also with thorax CT images and it was concluded that patients with high NLR and PLR values should undergo CT examination earlier since these patients have higher rates of mortality and need more intensive care admission **[21,22]**. It was stated that SII is a promising marker in the evaluation of disease activity during dermatologic involvement of Behçet’s disease **[23]**. It was also found that in psoriatic patients it positively correlated with PASI values **[24]**. In the present study, SII and PLR values were higher in IgG4-RD than the control group. In IgG4-RD patients, in the comparison between these indices, SII was found to be more diagnostic than NLR while no significant difference was found between SII, SIRI and PLR values in terms of prediction of IgG4-RD (p = 0.08; p = 0.4; p > 0.05). The fact that SII was more diagnostic than NLR in IgG4-RD patients was attributed to the addition of platelets to this ratio. It was also found that SII, SIRI, and NLR values correlated with CRP but not with ESR in the IgG4-RD group. In conclusion, it was established that the SII value is more valuable index than others in that it predicts diagnosis and differential diagnosis and reflects disease activity more accurately. Lymphocyte values were found to be highest in the IgG4-RD group, while neutrophil value was highest in the GPA group. In GPA and sarcoidosis groups, all indices were found to be significantly higher than control group. SII cutoff values were found to be highest in GPA group, while they were similar in sarcoidosis and IgG4-RD groups. Cutoff value of all indices other than SII was found to be higher in sarcoidosis group than IgG4-RD group. Although SII by itself may be a supportive finding in discriminating GPA from IgG4-RD, other indices should also be evaluated so that they can be discriminate from sarcoidosis.

We believe that the use of all indices in clinical practice is effective ancillary testing for the differential diagnosis of diseases. Even though they are not used for diagnosis and predicting prognosis on their own, they may be used as a supportive method in addition to physical examination, radiological examination, and laboratory tests. In GPA, inflammatory indices have a higher correlation with sedimentation and CRP than IgG4-RD, indicating that GPA may have a more aggressive course, even if it has limited organ involvement. IgG4 disease has lower activity at the time of diagnosis than GPA or sarcoidosis and hence should not be initially considered in patients with high NLR, SII and SIRI values and in such patients GPA and subsequently, sarcoidosis should be ruled out in diagnostic process.

Basic hematologic indices might help to differentiate IgG4-RD from limited GPA and sarcoidosis. Moreover, combination of these indices may be helpful the physician in the assessment of IgG4-RD disease activity. Further research is required to establish how these indices can be used in the clinical setting to improve the clinical management of IgG4-RD.

There are several limitations in our study. Major drawback is its crosssectional design. We could not determine whether its levels changed with treatment and whether it may predict relapse. Maybe in the future we can determine how response levels to treatment change in IgG4-RD patients only with serial biomarker measurements.

## Figures and Tables

**Table 1:** Demographic and clinical features of patients.

		Groups				*p*

		IgG4-RD (n = 30)	Sarcoidosis (n = 46)	GPA (n = 38)	Control (n = 27)	

**Age**	Mean *±* SD	55.43 ± 14.0	53.5 ± 12.4	48.2 ± 15.2	42.6 ± 14.1	0.058*

**Sex, n (%)**	Female	13 (43.3)	32 (92.6)	22 (57.9)	15 (55.6)	0.152**
	Male	17 (56.7)	14 (30.4)	16 (42.1)	12 (44.4)	

**Smoking habits**	Current n, (%)	5 (23.8)	5 (15.6)	6 (23.1)	7 (25,9)	0.57***
	Former n, (%)	4 (19.0)	13 (40.6)	8 (30.8)	9 (33,3)	
	Never n, (%)	12 (57.1)	14 (43.8)	12 (46.2)	11 (40.7)	

**Region of involvement, n (%)**	Orbita	7 (24.1)	3 (6.5)	4 (10.5)		
	Paranasal sinus	-	2 (4.3)	25 (65.7)		
	Lung	7 (24.1)	34 (73.9)	10 (26.3)		
	Kidney	1 (3.4)	2 (4.3)	3 (7.9)		
	GI	-	-	-		
	Nervous system	-	-	-		
	Lacrimal/salivary	10 (33.3)	5 (10.8)	1 (2.6)		
	glands					
	RPF	5 (16.6)	-	-		

GPA: granulomatosis polyangitis, GI: gastrointestinal, RPF: retroperitoneal fibrosis.

* One-way ANOVA test and Bonferroni test,

** chi-square tests,

*** Fisher’s exact tests.

**Table 2:** Distribution of histopathological findings, comorbidities and treatment.

IgG4-RD (n = 30)		n (%)

**IgG4-related mass**	Present	20 (69.0)
	Absent	9 (31.0)

**Histopathological findings**	Storiform fibrosis	6 (28.6)
	Dense lymphoplasmocytic infiltrate	21 (70)
	Obliterative phlebitis	1 (4.8)
	Eosinophilia	5 (23.8)
	Fibrosis	14 (66.7)
	Necrosis	4 (19.0)

**Comorbidity**	Hypertension	10 (33.3)
	Chronic arterial disease	6 (20.0)
	Chronic renal disease	3 (10.0)
	Diabetes mellitus	2 (6.7)
	Cancer	3 (10.0)
	Asthma	2 (6.7)
	Chronic obstructive pulmonary disease	2 (6.7)

**Treatment**	Corticosteroid	15 (88.2)
	Methotrexate	17 (56.7)
	Rituximab	5 (16.7)
	Cyclophosphamide	2 (6.7)
	Cyclosporine	2 (6.7)
	Follow-up without treatment	1 (7.7)

**Table 3:** Evaluation of laboratory measurements by groups.

Hematological biomarkers	Groups				*p*
	IgG4-RD (n = 30)	Sarcoidosis (n = 46)	GPA (n = 38)	Control (n = 27)	
**SII (**×**10^9^/L)** *median (min-max)*	572 (102––5583)^a^	855 (223––7103)^a^	1228 (36.1––7642)^b^	434 (172––897)^c^	**0.001** **
**SIRI (**×**10^9^/L)** *median (min-max)*	1.2 (0.1–12.9)^a^	2 (0.6–27)^b^	2.1 (0.1–21.4)^b^	0.9 (0.4–2.2)^a^	**0.001** **
**NLR** *median (min-max)*	2 (0–11.7)^a^	2.8 (1–14.8)^b^	4.1 (0.3–18.3)^c^	1.7 (1–3.1)^a^	**0.001** **
**PLR** *median (min-max)*	130 (56.8–546)^a^	195 (63.2–388)^b^	216 (50.2–500)^b^	104 (57.5–253)^c^	**0.001** **
**MPV (fL)** *median (min-max)*	8.7 (6.3–11)^a^	8.9 (6.2–14.1)^a^	9.1 (6.2–11.4)^a^	9.4 (7.5–11)^a^	0.362***
**RDW (%)** *median (min-max)*	15.2 (12.3–27.6)^a^	13.7 (12–20.2)^b^	14.7 (11.8–20.7)^a^	13.5 (12.2–16.1)^b^	**0.009** **
**Eosinophil (**×**10^9^/L)** *median (min-max)*	0.2 (0–5.3)^a^	0.1 (0–0.6)^a^	0.1 (0–0.5)^a^	0.1 (0–0.6)^a^	0.064**
**Platelet (**×**10^3^/uL)** *median (min-max)*	299 (162–819)^a^	294 (190–499)^a^	323 (143–1126)^a^	263 (138–344)^a^	0.065**
**Lymphocyte (**×**10^3^/uL)** *median (min-max)*	2.3 (0.7–3.8)^a^	1.5 (0.5–3.8)^b^	1.5 (0.6–2.9)^b^	2.4 (1.2–3.5)^a^	**0.001** **
**Neutrophil (**×**10^3^/uL)** *median (min-max)*	4.9 (1.8–14.7)^a^	4.5 (2.5–20.1)^a^	6.2 (0.7–20.1)^b^	3.8 (2.6–7.3)^a^	**0.001** **
**Monocytes (**×**10^3^/uL)** *median (min-max)*	0.5 (0.1–1.2)^a^	0.6 (0.4–9.1)^a^	0.6 (0.3–1.5)^a^	0.6 (0.3–0.9)^a^	0.080**
**ESR (mm/h)** *median (min-max)*	51 (5–118)^a^	34 (6–118)^a^	45 (9–140)^a^	11 (2–23)^b^	***0.001*** **
**CRP (mg/L)** *median (min-max)*	14 (2.2–393)^a^	8.1 (1.4–104)^a^	18.3 (1–317)^a^	2.65 (1–6.79)^b^	***0.002*** **

CRP: C-reactive protein, ESR: erythrocyte sedimentation rate, MPV: mean platelet volume, NLR: neutrophil to lymphocyte ratio PLR: platelet to lymphocyte ratio, RDW: red cell distribution width, SII: systemic inflammatory index, SIRI: systemic inflammatory response index. There are no difference between groups with the same letter such as while there is no statistical difference between those with the letter (a–a), (b–b), (c–c), there is a statistical difference between (a–b), (a–c), and (b–c).

** Kruskal-Wallis test and Dunn’s test,

*** one-way ANOVA test and Bonferroni test.
